# Exercise inhibits doxorubicin-induced cardiotoxicity by preventing cardiac and endothelial cell senescence by activating FNDC5/Irisin signaling

**DOI:** 10.21203/rs.3.rs-10910107/v1

**Published:** 2026-09-29

**Authors:** Prince Jeyabal, Pradeep Shrestha, Yuanzheng Yang, Fei Wang, Zhanglong Peng, You Zhou, Yan Zheng, Eugenie S. Kleinerman

**Affiliations:** 1. Division of Pediatrics Research, The University of Texas MD Anderson Cancer Center, Houston, TX 77030, USA.; 2. Department of Cancer Biology, The University of Texas MD Anderson Cancer Center, Houston, TX 77030, USA.

**Keywords:** Doxorubicin, cellular senescence, cardiotoxicity, exercise, FNDC5/Irisin

## Abstract

Exercise’s effect on Dox-induced cardiac senescence in the heart was investigated using p16–3MR transgenic mice. Mice treated with doxorubicin (Dox) had decreased cardiac function, increased p16INK4a+ and b-Galactosidase+ senescent cells in the heart, decreased HMGB1 and calreticulin expression, endothelial cell senescence, and decreased TUJ1 expression (indicating reduced axonal density), increased expression of Semaphorin3A and p-cofilin with impaired F-actin depolarization and cytoskeleton disorganization. None of these changes were seen in mice treated with Dox + Exercise. Exercise sustained the levels of FNDC5 in the heart compared with the Dox-treated hearts suggesting a link between exercise, decreased senescence, and the FNDC5/ irisin pathway. In vitro studies using human cardiomyocytes and endothelial cells confirmed that irisin attenuated Dox-induced senescence. Taken together, these data provide evidence that exercise mitigates Dox-induced cardiotoxicity by suppressing cardiovascular senescence via a mechanism involving the exercise-induced myosin irisin, and that exercise is a potential intervention for preventing Dox-induced cardiotoxicity.

## Introduction

The increased incidence of cardiovascular diseases, and its effect on both morbidity and mortality have made a significant impact on healthcare expenditures^1,2^. Among adolescent and young adult (AYA) cancer survivors ~46% have experienced at least one cardiovascular disease-related symptom by the age of 50. By contrast cardiomyopathies in the control population were only 15.7%^3,4^. Doxorubicin (Dox), a chemotherapy agent used to treat multiple solid tumors including sarcomas in pediatric and AYA patients, is associated with dose-limiting cardiotoxicity. Significant decline in left ventricular ejection fraction has been observed in patients following Dox treatment^5,6^. Numerous potential mechanisms have been proposed to be involved in Dox-induced cardiotoxicity, including oxidative stress with the generation of ROS, and induction of cardiac cell senescence. Cellular senescence is a biological process characterized by the loss of cellular replication potential and increased cellular dysfunction. Senescent cells remain metabolically active and accumulate with age in tissues throughout the body, including the cardiovascular system^7,8^. *Cardiac* senescence refers to the process of aging in the heart, and has been shown to play a key role in the development of various cardiovascular diseases and the decline in heart function^9–11^. Recent studies demonstrated that Dox accelerates cellular senescence^12,13^. In mice treated with Dox there was an increased number of senescence cells in the heart after therapy. Removal of these senescent cells prevented the development of Dox-induced cardiac dysfunction^12^, supporting a role for senescence in Dox-induced cardiotoxicity^12,13^. Other studies have shown that senescent cells are increased in the hearts of mice and humans with heart failure, and that the elimination of senescent cells can improve cardiac function and reduce the risk of heart disease^14,15^.

Numerous studies have implicated oxidative stress, a pro-inflammatory microenvironment, and the accumulation of DNA damage as key drivers of the senescence process in the heart^16–18^. Indeed, the cardiotoxic effects of Dox were potentiated by increasing its pro-senescent effects without inducing apoptosis^19^. This in turn led to the activation of a range of cellular pathways, including p16^INK4a^ pathways, that promote premature cellular senescence^13,20^. The accumulation of senescent cells not only promotes the adverse cardiac effects of chemotherapy but also drives the progression of a variety of other pathologies including cancer relapse^12,21^.

Given the major problem of cardiovascular disease in childhood and AYA cancer survivors, there has been increasing interest in developing interventions to prevent and reduce the risk of heart disease in this population. We previously showed that exercise during Dox therapy prevented Dox-induced cardiac damage, the induction of autophagy and mitophagy in the cardiac cells, cardiac fibrosis, and the decline in functional activity^22–25^. Exercise during Dox also preserved cardiac blood flow and vascular morphology. Exercise initiated after therapy promoted cardiac repair and the recovery of cardiac function^24^. Given the potential role of senescence in Dox-induced cardiotoxicity, we wished to determine whether exercise mediated its protective effect by decreasing Dox-induced senescence in the heart using our juvenile mouse cardiotoxicity model in p16–3MR transgenic mice. We sought to determine whether exercise prevented the development of cardiac cellular senescence and whether inhibiting the cellular senescence correlated with its ability to protect the heart against Dox-induced damage. We also investigated a potential mechanism by which exercise inhibits the senescence process. Identifying potential mechanisms by which exercise decreases chemotherapy-induced cardiac senescence will allow the development of novel drug therapies to prevent and decrease chemotherapy-induced cardiomyopathies. This in turn can be expected to improve longevity and quality of life for childhood and AYA cancer survivors where cardiac disease is one of the major health risks.

## Results

### Exercise inhibits Dox-induced cardiotoxicity and senescence of cardiomyocytes, endothelial cells, and fibroblasts

The p16–3MR transgenic mouse model^12,26^ allows the identification and quantification of senescent cells in the heart. We demonstrated that, similar to our findings in wild-type mice^22,25^, 2 weeks of Dox therapy resulted in a significant decrease in both EF and FS 24 hours after therapy (Supplementary Fig. 1A, B). This decreased function was accompanied by an increase in senescent cells, as quantified by an increase in p16-RFP^+^ cells (Supplementary Fig. 1C). The ability of Dox to induce cardiomyocyte senescence was confirmed in vitro. Enhanced b-Galactosidase activity is one of the characteristics of senescent cells^26^. Following incubation of neonatal mouse and human cardiomyocytes with Dox, there was a significant increase in b-Galactosidase^+^ cells (Supplementary Fig. 1D, E) and down-regulation of HMGB1 and Calreticulin (Supplementary Fig. 1F, G) another hall mark of cellular senescence. In senescent cells, the intracellular level of HMGB1 and Calreticulin decrease due to secretion into the extracellular space^27,28^.

Having confirmed that Dox therapy resulted in a decrease in EF and FS in p16–3MR mice similar to wild-type mice, with an increase in cellular senescence, we next investigated the effect of exercise on Dox-induced senescence. P16–3MR transgenic mice were treated with PBS, Dox or Dox+Ex. Cardiac function was evaluated using echocardiography 2 weeks after Dox-treatment (Supplementary Fig. 2A, B). Once again there was a significant decrease in both EF and FS in p16–3MR mice treated with Dox (Fig. 1A, B, and Supplementary Fig. 2A, B). By contrast there was no statistical difference between the control and Dox+Ex-treated mice. Following echocardiography mice were euthanized to obtain heart tissue for analysis. There was a significant increase in p16-RFP^+^ cells in the hearts of Dox-treated mice (p<0.0001), but not in the Dox+Ex-treated mice (Fig. 1C). Decreased intracellular levels of HMGB1 and Calreticulin are expected in senescent cells. Indeed, analysis of cardiac tissue showed that exercise also inhibited the Dox-induced decrease in HMGB1 (Fig. 1D–G) and calreticulin (Fig. 1 H–K) as quantified by immunofluorescent staining and western blot analysis. Taken together, these results indicate that exercise prevented both Dox-induced cellular senescence and cardiac functional decline.

Coronary vascular endothelial cells are important for vessel integrity and function. These vessels play a critical role in bringing oxygen and nutrients to the cardiac tissue, thereby maintaining the optimal tissue microenvironment. The senescence of vascular endothelial cells can result in compromised vessels. We previously showed that following Dox therapy the cardiac vascular structure was altered with a change in vascular morphology and a decrease in CD31^+^ endothelial cells, leading to decreased cardiac blood flow^24^. We also showed that exercise prevented these vascular changes and preserved cardiac blood flow^23,24^. We therefore investigated whether *endothelial cell senescence* was seen following Dox therapy, and whether exercise prevented Dox-induced senescence in the vascular endothelial cells. Using CD31 as the endothelial cell marker we quantified the number of p16/CD31 double positive cells in the heart sections from the different treatment groups. As shown in Fig. 2A, there was a significant increase in the number of senescent p16/CD31 double positive cells in the Dox-treated hearts compared to control (p<0.0001). This increase was not seen in hearts from the Dox+Ex-treated mice. There was no significant difference between control and Dox+Ex-treated mice. These data indicate that Dox therapy induced senescence of the cardiac endothelial cells, and that exercise inhibited the Dox-induced endothelial cell senescence.

We next evaluated the effect of Dox on the cardiomyocyte and fibroblast cell populations in the heart sections. Troponin I expression was used as a marker of cardiomyocytes, vimentin as a marker of fibroblasts, and p16 (RFP-tagged) as a senescence marker. Senescent cardiomyocytes were identified by colocalization of p16 and troponin. Quantification of p16/troponin double positive cells showed a significant increase in the number senescent cardiomyocytes in the Dox-treated mice (p<0.01), but not the Dox+Ex-treated mice compared to the control mice (Fig. 2B). There was no statistical difference between the control mice and mice treated with Dox+Ex. Finally, fibroblast senescence was quantified using vimentin (a fibroblast marker) in combination with p16 expression. Immunofluorescence analysis revealed that Dox therapy significantly increased the percentage of p16/vimentin double-positive cells (Fig. 2C; p<0.05). By contrast there was no difference in the number of senescent fibroblasts in the hearts from Dox+Ex-treated mice compared with the control mice. These data indicate that exercise prevented Dox-induced senescence of endothelial cells, cardiomyocytes, and fibroblasts.

### Exercise prevented Dox-induced reduction in axonal density

As shown in Fig. 2A, Dox therapy resulted in the senescence of endothelial cells in the heart. Semaphorin-3A (Sema3a) is a molecular link between endothelial cell senescence and progressive cardiac denervation. Vascular aging and senescent endothelial cells promote the release of Sema3a, which results in the decreased density of neuronal axons and the prevention of axonal innervation^29^. This in turn leads to cardiac electrical instability and arrhythmias thru which is also associated with decreased cardiac reparative function. Neuron-specific class III beta-tubulin (TUJ1) is the marker that identifies the neuronal axons. Elevated levels of Sema3a lead to a decrease in axons. We therefore next investigated whether Dox-induced cardiac endothelial cell senescence correlated with changes in Sema3a and TUJ1 expression using western blot (Fig. 3A–C) and immunofluorescent staining (Fig. 3 D–F). As expected, both western blot and mean immunofluorescence intensity showed that Sema3a expression was significantly increased (p<0.05) and TUJ1 significantly decreased (p<0.05) in the hearts from the Dox-treated but not the Dox+Ex-treated mice. The decreased expression of TUJ1 in the Dox-treated hearts indicates a reduction in axon density. By contrast, the TUJ1 mean fluorescent intensity in the Dox+Ex-treated hearts was not significantly different than the controls, indicating that exercise inhibited the Dox-induced reduction in cardiac axonal density. These data indicate that Dox-induced endothelial cellular senescence correlated with decreased neuronal axon density, further suggesting that exercise protected against this effect on neuronal axons by inhibiting Dox-induced endothelial cell senescence.

### Effect of Dox and Dox+Ex on the actin cytoskeleton, F-actin polarization and impaired depolymerization by cofilin phosphorylation

The actin cytoskeleton, particularly F-actin, is an essential element of the contractile machinery in cardiomyocytes^30,31^. F-actin is critical for effective cardiac contraction and function as it provides structural support for the cardiac muscles. While loss or translocation of HMGB1 and calreticulin from the nucleus is a hallmark of cellular senescence, these nuclear envelope proteins also play a pivotal role in modulating actin cytoskeletal dynamics, and their decreased expression can have an effect on the cytoskeletal structure of the heart. We therefore next evaluated the effect of Dox and Dox+Ex on the actin cytoskeleton. Confocal images of F-actin distribution in control, Dox-treated, and Dox+Ex-treated mouse hearts demonstrated disruption of F-actin organization in the hearts from the Dox-treated mice compared to the control group (Fig. 3G). Dox-induced F-actin disruption was not seen in the hearts from the Dox+Ex-treated mice indicating a protective effect of exercise against Dox-induced F-actin disruption. Next, we evaluated the expression of phosphorylated cofilin which has been shown to impair F-actin depolymerization and lead to F-actin disruption^32,33^. Dox treatment significantly increased the expression of phosphorylated cofilin (p-cofilin) in the heart with an increase in the p-cofilin/histone intensity (Fig. 3H; p<0.05). This increase was not seen in the Dox+Ex mice confirming the protective effect of exercise against Dox-induced disruption of F-actin, which supports that the effect of Dox on F-actin is linked to the induction of senescence.

### Exercise prevented Dox-induced senescence via the FNDC5/irisin axis

Fibronectin type III domain–containing 5 (FNDC5), a type I transmembrane glycoprotein, is a precursor protein that when proteolytically cleaved releases the exercise-induced myokine *irisin*, which confers cardio-protection for diverse pathological cardiomyopathies^34–36^. Irisin ameliorates age-associated cardiac decline by actively suppressing cardiac senescence^37,38^. This suggests that the FNDC5/irisin molecular pathway may be involved in the mechanism by which exercise prevents Dox-induced cellular senescence and preserves cardiovascular function.

To explore the association between exercise-protection, the FNDC5/irisin pathway, and cardiomyocyte and endothelial cell senescence, human cardiomyocytes (HCMs) were treated with Dox for 24h, and then harvested 7 days later. Analysis using the senescence marker p16 showed that Dox treatment induced the upregulation of p16 and the downregulation of FNDC5 (Fig. 4A–C). To confirm these in vitro effects in vivo, we analyzed the FNDC5 expression in the heart tissues from the p16–3MR transgenic mice described in Fig. 1C, where there were increased p16^+^ cells in the Dox-but not the Dox+Ex-treated mice. Immunofluorescent staining and western blot analysis showed a significant reduction in FNDC5 in the hearts from the Dox-treated mice compared to controls (Fig. 4D–F). By contrast the FNDC5 expression in the hearts from the Dox+Ex-treated mice was not significantly different from controls. We next explored whether the FNDC5/irisin pathway can be leveraged to decrease Dox-induced cardiac senescence using irisin protein. Human cardiomyocytes (HCMs) and human coronary artery endothelial cells (HCECs) were treated with Dox with or without irisin. There were significantly fewer β-galactosidase^+^ cells in the HCMs (Fig. 5A) and HCECs (Fig. 5B) treated with Dox+irisin. Using immunofluorescence (Fig. 5C, D) and western blot analysis (Fig. 5E–G) we demonstrated that Dox therapy increased the number of p16^+^ senescent cells in the heart and also upregulated Sema3a. Irisin inhibited these changes. Irisin also inhibited the upregulation of p-cofilin (Fig. 5H, I). Taken together, these data support a link between the FNDC5/irisin pathway, and the ability of exercise to inhibit Dox-induced cellular senescence and cardiotoxicity.

In summary, our findings support the hypothesis that Dox therapy induced cardiac, fibroblast, and endothelial cell senescence, resulting in the upregulations of Sema3a and cofilin phosphorylation. This leads to F-actin disruption and decreased axonal density. These senescence-induced changes in F-actin and axon density are likely to contribute to Dox-induced cardiotoxicity and the subsequent development of various cardiomyopathies including arrhythmias. Exercise during Dox prevented the induction of cellular senescence in the heart and changes in the actin skeleton, and preserved axonal density. Our data suggests that the protective effect of exercise with inhibition of cardiac cell senescence is mediated through FNDC5/irisin signaling and the release of irisin as depicted in Fig. 6.

## Discussion

Cancer survivors have an increased risk for developing myopathies and cardiomyopathies at an early age. Children, adolescents, and young adults (AYAs) with sarcoma are particularly vulnerable, as high doses of Dox are used for treatment. Dox has been shown to induce cardiac cell senescence and an accelerated aging phenotype. Therefore, interfering with Dox-induced senescence may impact the development of cardiac disease. Using our juvenile mouse cardiotoxicity model we previously reported that exercise during Dox prevented acute cardiac damage and functional decline, and enabled superior recovery following myocardial ischemia^23,24^. Because of the link between Dox-induced cardiac senescence and the development of cardiotoxicity, we sought to determine whether the protective effect of exercise was mediated by blocking the development of senescence. For these studies we elected to use p16–3MR transgenic mice, in order to identify and quantify senescent cells in the heart. Mice treated with Dox showed decreased cardiac function, (as quantified by EF and FS via parasternal long axis echocardiography). The decreased cardiac function was associated with increased p16^+^β-galactosidase^+^ cells in the heart, and a reduction in HMGB1 and calreticulin, all signs of Dox-induced cellular senescence. There was also disruption of F-actin and increased p-cofilin in the heart tissues. Our findings align with previous studies showing that Dox-induced cardiac cell senescence contributes to cardiotoxicity^13,39^.

By contrast in the mice treated with Dox+Ex cardiac function was maintained and there was no increase in p16^+^ or β-galactosidase^+^ cells in the heart. The levels of HMGB1, calreticulin, and p-cofilin were not significantly different from control levels and there was preservation of F-actin organization. These data show a correlation between cellular senescence and the development of cardiac damage. In addition, the data indicates that exercise blocks cellular senescence thereby preserving cardiac function. These data, once again, link the process of senescence with the subsequent development of cardiotoxicity.

In addition to inhibiting cardiomyocyte senescence, exercise during Dox also decreased the senescence of cardiac *endothelial cells* and *fibroblasts*. This is significant as we previously showed that Dox treatment altered the morphology of the cardiac vasculature by decreasing vascular endothelial cells resulting in collapsed vessels and decreased blood flow^23,24^. These vascular changes and decreased cardiac blood flow were not seen in the Dox+Ex mice. Demonstrating that exercise prevented endothelial cell senescence provides a mechanism by which exercise prevented the Dox-induced damage to the cardiac vessels, and maintained cardiac vascular morphology and blood flow in the mice treated with Dox+Ex. Maintaining normal cardiac vascular endothelial coverage with open vessel lumens and adequate blood flow is expected to maintain tissue oxygen levels and the delivery of nutrients, which in turn may impact the ability of the cardiac tissue to recover and heal. These findings may therefore explain the improved recovery of mice treated with Dox+Ex after an ischemia episode^24^. Our previous studies showed that survival at 100 days post-MI for mice treated with Dox was 22%, compared to 56% in mice receiving Dox+Ex, and that this survival rate was not significantly different from the survival rate of control mice that had received no prior therapy. Other investigations have shown that Dox-induced *endothelial cell* damage and dysfunction play a critical role in Dox-induced cardiotoxicity^40^. Inhibiting endothelial cell senescence using the pharmacologic agent ABT 263, which targets BCL-2, decreased Dox-induced ventricular endothelial dysfunction^41^. Our studies are the first to link Dox-induced endothelial cell *senescence* with functional cardiotoxicity and the protective effect of exercise in inhibiting endothelial cell senescence.

We also showed that exercise prevented a decrease in TUJ1-positive cells, preserving axonal density. Therefore, reducing the senescence of cardiac endothelial cells and maintaining normal blood flow and neuronal axon density may preserve the cardiac microenvironment that is essential for electrical stability. In addition to preserving vascular morphology and blood flow, exercise inhibited the development of cardiac fibrosis and collagen deposition following Dox^24^. Cardiac fibrosis arises from the deposition of extracellular matrix proteins such as collagen. Cardiac fibroblasts participate in formation of the fibrotic tissue which can be initiated by the induction of senescence in the cardiac fibroblasts. Eliminating or blocking the senescence of cardiac fibroblasts may therefore be responsible for the reduced collagen deposition and absence of fibrosis in the hearts from the Dox+Ex mice in our previous study^24^.

This study also presents a potential mechanism by which exercise prevents Dox-induced cardiac senescence. FNDC5, a cardioprotective factor that is essential for resistance to cardiotoxic insults, has been shown to be downregulated by Dox, and decreased in aged murine myocardium^34^. These findings support the correlation that we showed between the induction of senescence and decreased FNDC5 in the hearts following Dox treatment. FNDC5 interferes with Dox-induced cardiac damage by blocking oxidative stress and mitochondrial cell death^34^. Furthermore, the downregulation of FNDC5 in myocardium has been shown to trigger fibrosis, compromise ventricular function, and increase the cardiotoxic effects of Dox^38,42^. By contrast, FNDC5 overexpression prevented myocyte senescence^38^ and was cardioprotective against Dox-induced cardiac damage^42,43^. We demonstrated that Dox therapy resulted in cardiac cellular senescence (Fig. 1C), decreased FNDC5 in the heart (Fig. 4D), cardiac fibrosis^24^ and decreased cardiac function (Fig. 1 A, B). By contrast, exercise prevented cardiac cellular senescence (Fig. 1C), preserved the levels of FNDC5 (Fig. 4D) and cardiac function (Fig. 1A, B), and inhibited the development of fibrosis^24^. Taken together these data support our hypothesis that the cardioprotective effect of exercise is linked to its ability to block the senescence process in the heart via the FNDC5 pathway. We further demonstrated that the exercise-induced myokine *irisin*, a hormone produced by muscles during physical activity which is cleaved from its precursor protein FNDC5, prevented the senescence of human cardiomyocytes and human cardiac endothelial cells, and the upregulation of SEMA3a and p-cofilin, two markers directly linked to endothelial cell senescence. We hypothesize that in addition to inhibiting Dox-induced *cardiomyocyte* senescence, irisin secretion (in response to exercise), may be responsible for the decreased cardiac *endothelial cell* senescence in the hearts of the mice treated with Dox+Ex thereby preserving vascular integrity and blood flow. In support of this hypothesis are the findings that irisin rescued diabetic cardiac vascular injury and improved endothelial function in diabetes^44^ and inhibited Dox-induced perivascular fibrosis via its effect on endothelial cells^45^.

It is important to note that the mouse model used to investigate the role of senescence in Dox-induced cardiotoxicity, and the ability of exercise to prevent cardiomyocyte, endothelial cell, and fibroblast senescence was in *juvenile* mice. This supports the relevance of our findings to what occurs in children and AYAs with sarcoma who receive high doses of Dox (>250 mg/m2). We have already demonstrated that our findings of early compromised diastolic function in this mouse model^24^ were similar to our clinical findings in both retrospective and prospective studies in AYA sarcoma patients^46,47^. Of the 56 AYA patients studied prospectively, 44% showed signs of diastolic dysfunction 1 year after diagnosis^47^. It is imperative to employ relevant preclinical models in mice of the appropriate age to evaluate potential interventions, define their mechanisms of action, and justify clinical trials for inhibiting and decreasing Dox-induced cardiotoxicity. The studies presented here support the contribution of Dox-induced senescence to the development of cardiac structural damage, cardiac vascular changes, and decreased heart function, and demonstrate the ability of exercise on to inhibit these Dox-induced toxicities. We demonstrated that Dox induced the senescence of not only cardiomyocytes, but also vascular endothelial cells and fibroblasts, which resulted in a disrupted actin cytoskeleton. Such changes in the cytoskeleton have been associated with an increased risk of cardiotoxicity. We demonstrated that exercise during therapy prevented cardiac and endothelial cell senescence and preserved the actin cytoskeleton and neuronal axon density. This data further confirms that exercise prevents Dox-induced cardiac damage, and suggests its protective effect occurs through inhibiting senescence in cardiomyocytes and vascular endothelial cells. Our studies indicate the potential of employing exercise as an intervention to prevent premature cardiac senescence following Dox therapy, in order to limit acute Dox-induced cardiotoxicity and the subsequent development of cardiac morbidities in AYA survivors. We also identified a potential pathway which is induced by exercise which can be targeted as an alternative to physical activity.

## Methods

### In vitro study design

Mouse neonatal cardiomyocytes were isolated and plated in culture dishes in a growth medium consisting of DMEM/F12, supplemented with 10% fetal bovine serum, 1% penicillin/streptomycin, and 1% Glutamax. The cells were allowed to adhere for 24 hours prior to treatment.

Human cardiomyocytes (HCMs) were cultivated in the medium enriched with 5% fetal bovine serum (FBS), 1% cardiac myocyte growth supplement (CMGS), and 1% penicillin/streptomycin solution (P/S). Including the HCMs all these items were procured from ScienCell Research Laboratories, Inc., located in Carlsbad, CA, USA. The cells were cultured in a controlled environment in an incubator set to 37 °C and a 5% CO_2_ atmosphere.

Primary human coronary artery endothelial cells (HCECs; ATCC, PCS-100–020) were cultured in Vascular Cell Basal Medium (PCS-100–030) supplemented with the Endothelial Cell Growth Kit (PCS-100–041) and 5% fetal bovine serum, according to the manufacturer’s instructions. Cells were maintained under standard culture conditions and used between passages 4 to 6 for all experiments.

Based on previous studies^12^ and our experimental optimization 250 nM Dox was used to induce cellular senescence. Higher concentration (1.25 μM or 2.5 μM) resulted in apoptosis. The cardiomyocytes were treated with 250 nmol/L Dox for 24 hours, after which they were washed, and the growth medium was changed. Seven days after the Dox treatment, the cells were analyzed for senescent markers using immunofluorescence staining and Western blot analysis.

### Senescence-associated β-galactosidase staining assay

In this assay, cultured cardiomyocytes were employed upon completion of the experiment. Right after the experiment, the cells were promptly fixed and subjected to an overnight staining process using an X-Galactosidase solution, utilizing a commercial Senescence β-Galactosidase staining kit (Cell Signaling). Following staining, the samples were carefully mounted and analyzed. Subsequently, images were captured at magnifications of 20X and 40X, and the resultant positive images of the β-Galactosidase staining were analyzed and graphed.

### Transgenic p16–3MR mice

Transgenic p16–3MR mice were obtained from the Jackson Laboratory (RRID: IMSR_JAX:037045). The p16–3R transgenic mouse model enables the identification of senescent cells in the heart by the 3MR (trimodality reporter) fusion protein, which consists of monomeric red fluorescent protein^48^. This promoter has previously been employed to track senescent cells in various mouse models^49–51^. All animal experiments were reviewed and approved by the Institutional Animal Care and Use Committee (IACUC) of The University of Texas MD Anderson Cancer Center.

Four weeks old p16–3MR mice were divided into two or three groups based on the experimental design. The mice were treated as follows: Control: PBS injection; Dox-only: 2.5 mg/kg Dox injected twice a week for 2 weeks; Dox+Ex: 2.5 mg/kg Dox injected twice a week for 2 weeks and simultaneous exercise consisting of walking for 45 minutes at a speed of 12 meters per minute 5 days a week for 2 weeks. Heart tissue was collected 2 weeks after the final treatment and processed for immunofluorescence and Western immunoblot analysis to evaluate the induction of senescence.

### Western blot analysis

For Western blot analysis, we collected cells and mouse heart tissues following treatment. Lysates were subsequently prepared from these samples and total proteins were extracted. Western blot analysis was conducted using specific antibodies, by established experimental conditions described as previously reported^23^.

### Immunofluorescence analysis

Cardiomyocytes or frozen tissue slides were prepared for Immunofluorescence analysis. The fixation and permeabilization of the frozen tissues were accomplished by immersing them in cold acetone for a duration of 10 minutes, in accordance with established procedures^23,52^. Subsequently, images of the stained tissues were acquired using a Leica DM5500 B upright and Leica DMi8 inverted microscope imaging system (Leica Microsystems, Buffalo Grove, IL, USA), then processed and analyzed utilizing Adobe Illustrator and Photoshop software (Adobe Inc., San Jose, CA, USA).

### Echocardiographic imaging and evaluation of cardiac function

Cardiac function was evaluated by echocardiography before and 2 weeks after the final Dox treatment using transthoracic echocardiography (Vevo 3100 echocardiography with a 40MHz linear signal transducer and 550D probe; VisualSonics, Toronto, CA). The parameters analyzed included left ventricular (LV) ejection fraction (EF) and fractional shortening (FS). For each mouse, at least five B-mode images were recorded, and EF and FS were calculated by Vevo Lab software.

### Statistical analysis

Data are presented as mean ± SEM. All experiments were performed using biological replicates as indicated in the figure legends. For comparisons between two groups, a two-tailed unpaired Student’s t-test was used. Comparisons among three or more groups were conducted using one-way analysis of variance (ANOVA) followed by Tukey’s multiple-comparisons test. Individual animals or independently cultured cell preparations were considered biological replicates. Sample sizes (n) for each experiment are indicated in the corresponding figure legends. Statistical significance was defined as P < 0.05. Analyses were performed using GraphPad Prism software (GraphPad Software, San Diego, CA, USA).

## Supplementary Material

1

## Figures and Tables

**Fig. 1:**
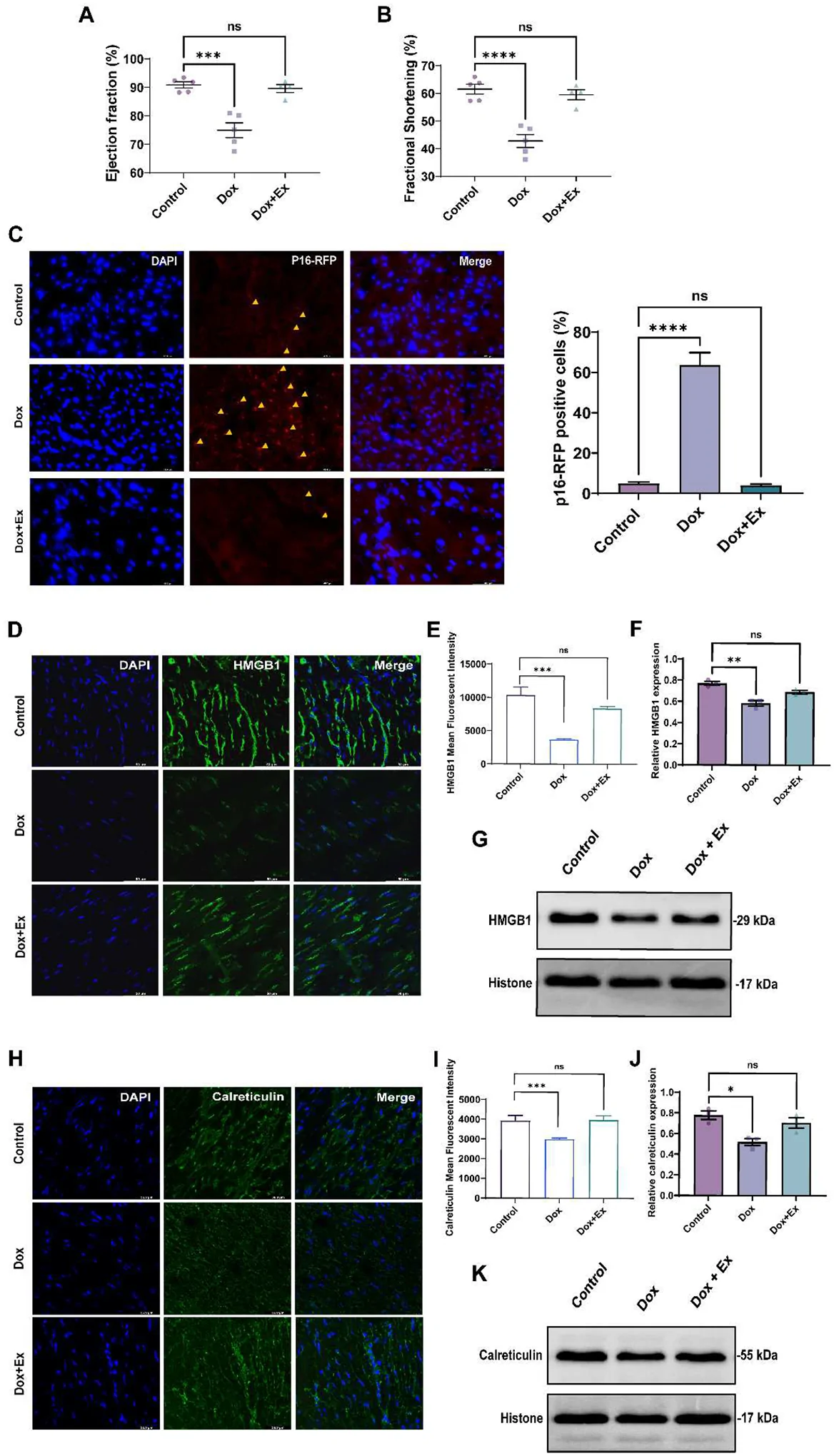
Exercise inhibits Dox-induced cardiac senescence and functional cardiotoxicity p16–3MR transgenic mice were treated with Dox or Dox+Ex for 2 weeks. Echocardiographic analysis of EF% (A) and FS% (B) was performed before and 2 weeks after therapy. Data are expressed as mean ± SEM; n=5 (C) Immunofluorescence analysis of p16-RFP accumulation in the hearts of control, Dox, and Dox+Ex-treated mice with quantification of p16-RFP positive cells (%). Scale bar = 48.6 μm. (D-G) HMGB1 and (H-K) calreticulin expression in the heart tissue. Representative immunofluorescence images of HMGB1 (D) and calreticulin (H) with the corresponding quantitative analyses (E and I). Protein levels were evaluated by western blotting (G and K) and quantified by densitometric analysis (F and J). Data are expressed as mean ± SEM; n=3. Scale bar = 50 μm. Statistical significance was determined using one-way ANOVA with multiple comparisons. Significance is denoted by *p ≤ 0.05, **p ≤ 0.01, ***p ≤ 0.001, and ****p ≤ 0.0001; ns, not significant.

**Fig. 2:**
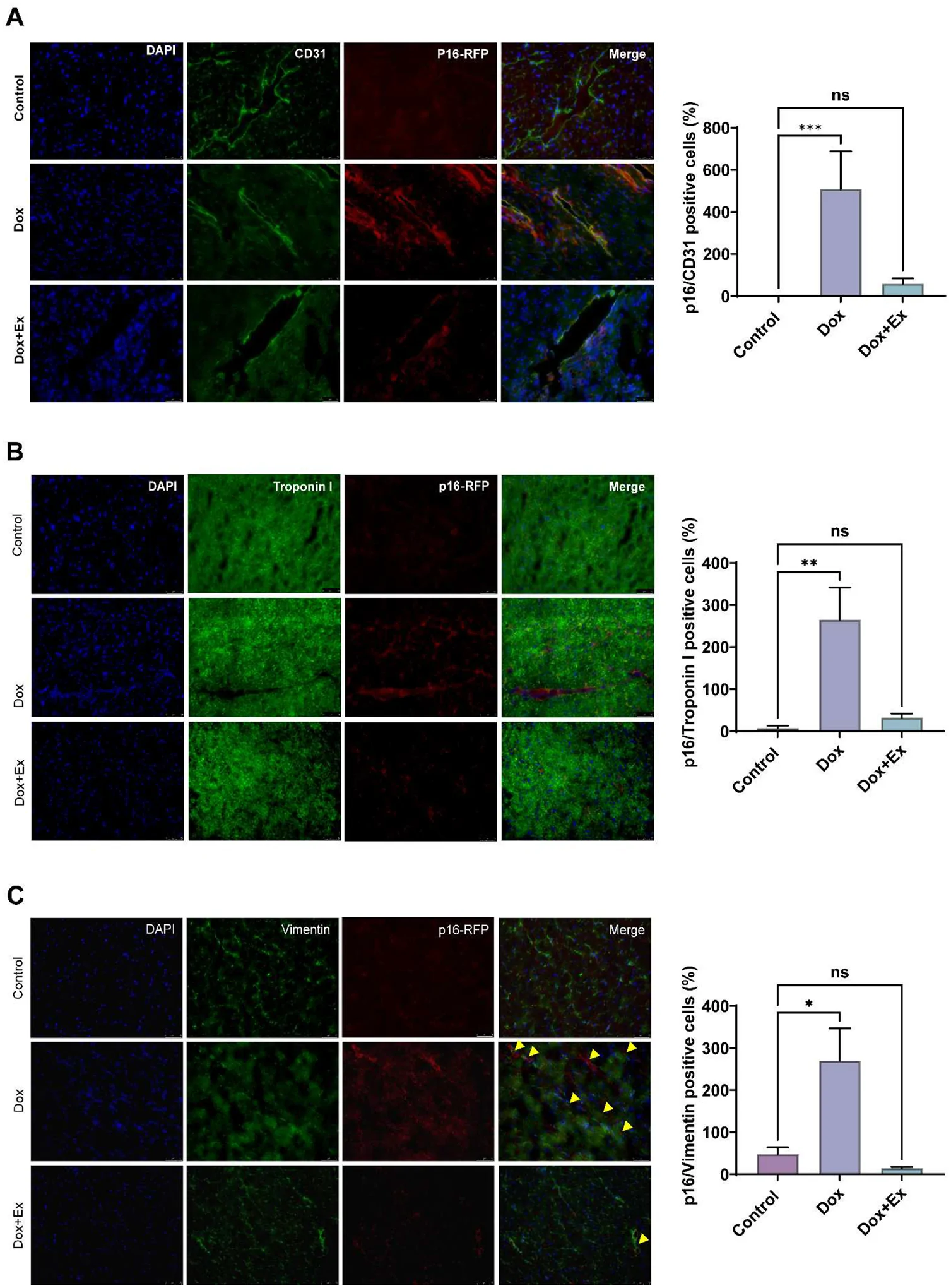
Effect of exercise on Dox-induced endothelial cell, cardiomyocyte and fibroblast senescence. Heart sections were analyzed for senescent endothelial cells (A), cardiomyocytes (B) and fibroblasts (C). (A) Senescent endothelial cells were identified and quantified using immunofluorescence and the colocalization of CD31 (green) and p16 (red). (B) Colocalization of the cardiomyocyte marker Troponin I (green) and p16 (red) were used to identify and quantify senescence cardiomyocytes. (C) Senescent fibroblasts were identified by colocalization of vimentin (green) and p16 (red). Scale bar = 50 μm. Data are expressed as mean ± SEM; n=5. Statistical significance was determined using one-way ANOVA with multiple comparisons. Significance is denoted by *p ≤ 0.05, **p ≤ 0.01, and ***p ≤ 0.001; ns, not significant.

**Fig. 3:**
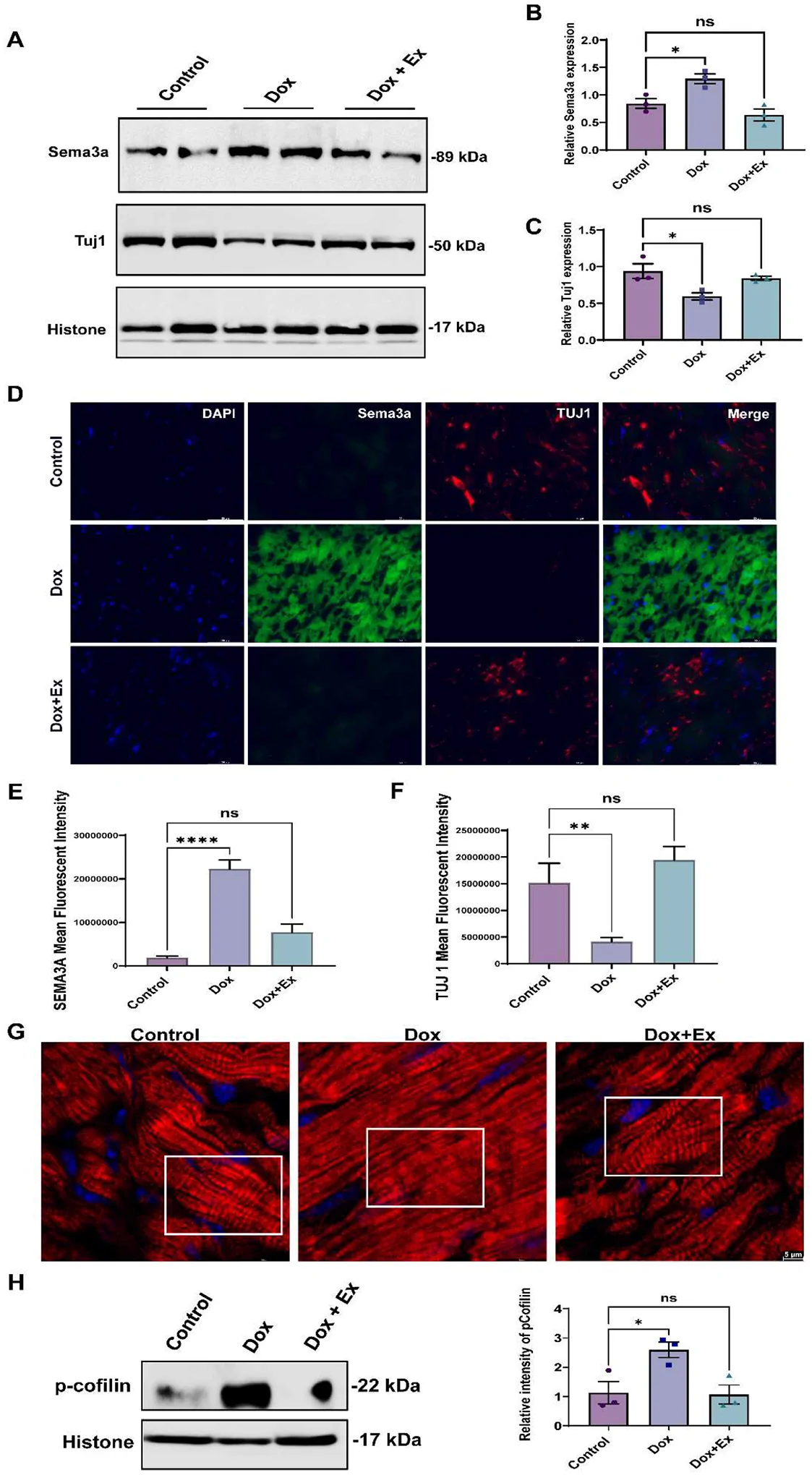
Exercise inhibits Dox-induced changes in Sema3a, TUJ1, F-actin histology and p-cofilin (A) Western blot analysis of Sema3a and TUJ1 expression in the heart (B, C) Corresponding densitometric quantification. Data are expressed as mean ± SEM; n=3 (D) Representative immunofluorescence images of Sema3a (senescent cell marker) (green) and TUJ1 (axonal marker), (red) in the heart. Scale bar = 50 μm. (E, F) Quantification of mean fluorescent intensity for Sema3a (E) and TUJ1 (F). Data are expressed as mean ± SEM; n=5. (G) Confocal images showing F-actin distribution in heart sections from control, Dox-treated, and Dox+Ex-treated mice. Scale bar = 5μm. (H) Western blot analysis and corresponding densitometric quantification of p-cofilin in cardiac tissue from control, Dox-treated, and Dox+Ex-treated mice. Histone was used as a loading control. Data are expressed as mean ± SEM; n=3. Statistical significance was determined using one-way ANOVA with multiple comparisons. Significance is denoted by *p ≤ 0.05, **p ≤ 0.01, and ****p ≤ 0.0001; ns, not significant.

**Fig. 4:**
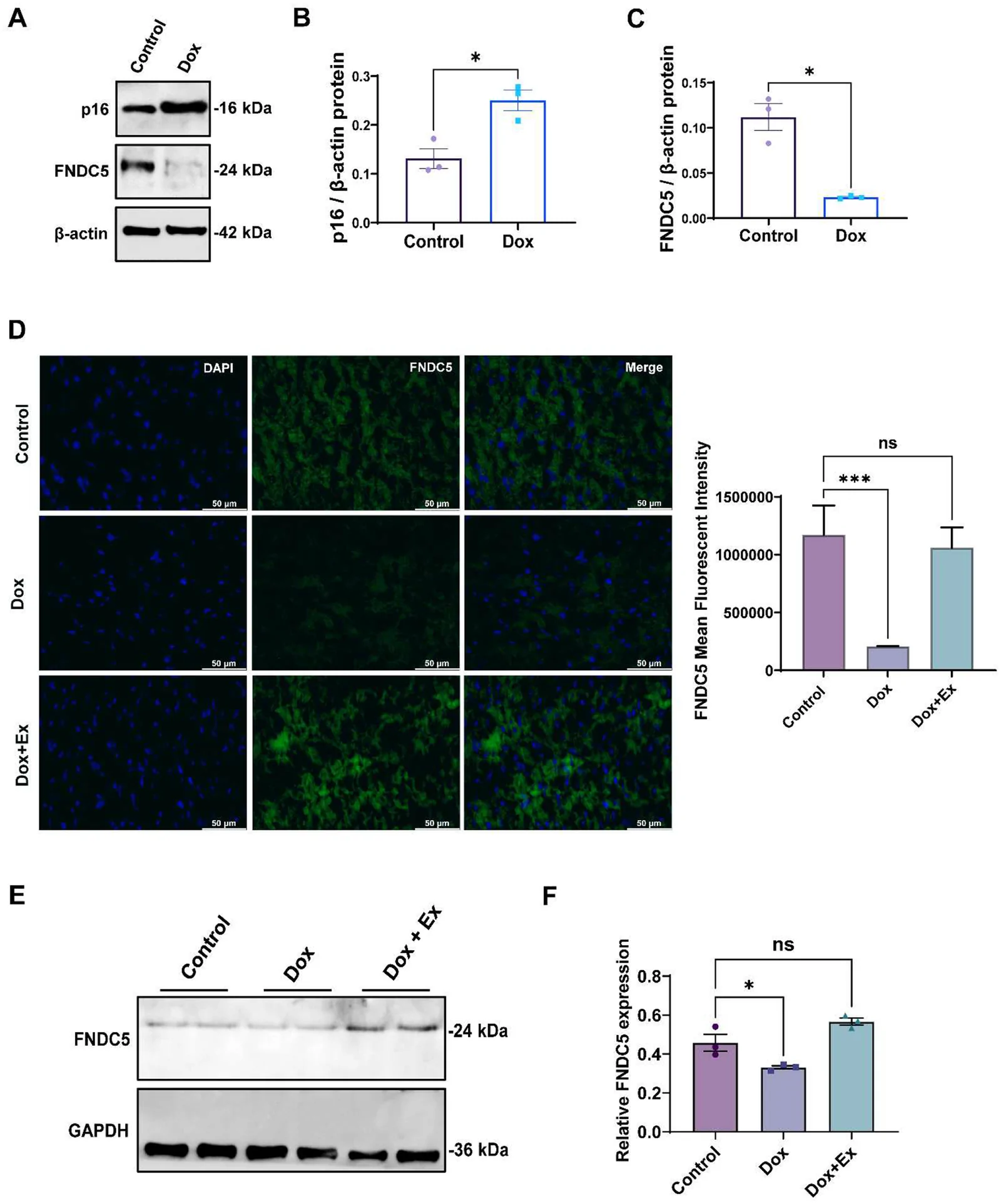
Exercise inhibits Dox-induced senescence and downregulation of FNDC5 Human cardiomyocytes (HCMs) were treated with Dox as outlined in experimental methods. (A) Expression of p16 and FNDC5. (B, C) Corresponding densitometric analysis. (D) Representative images of FNDC5 (green) expression, with quantification of FNDC5 mean fluorescent intensity. Scale bar = 50 μm. Data are expressed as mean ± SEM; n=5. (E, F) Western immunoblot analysis of FNDC5 protein expression in heart tissue (E) with densitometric analysis (F). Data are expressed as mean ± SEM; n=3. Statistical significance was determined using one-way ANOVA with multiple comparisons. Significance is denoted by *p ≤ 0.05, and ***p ≤ 0.001; ns, not significant.

**Fig. 5:**
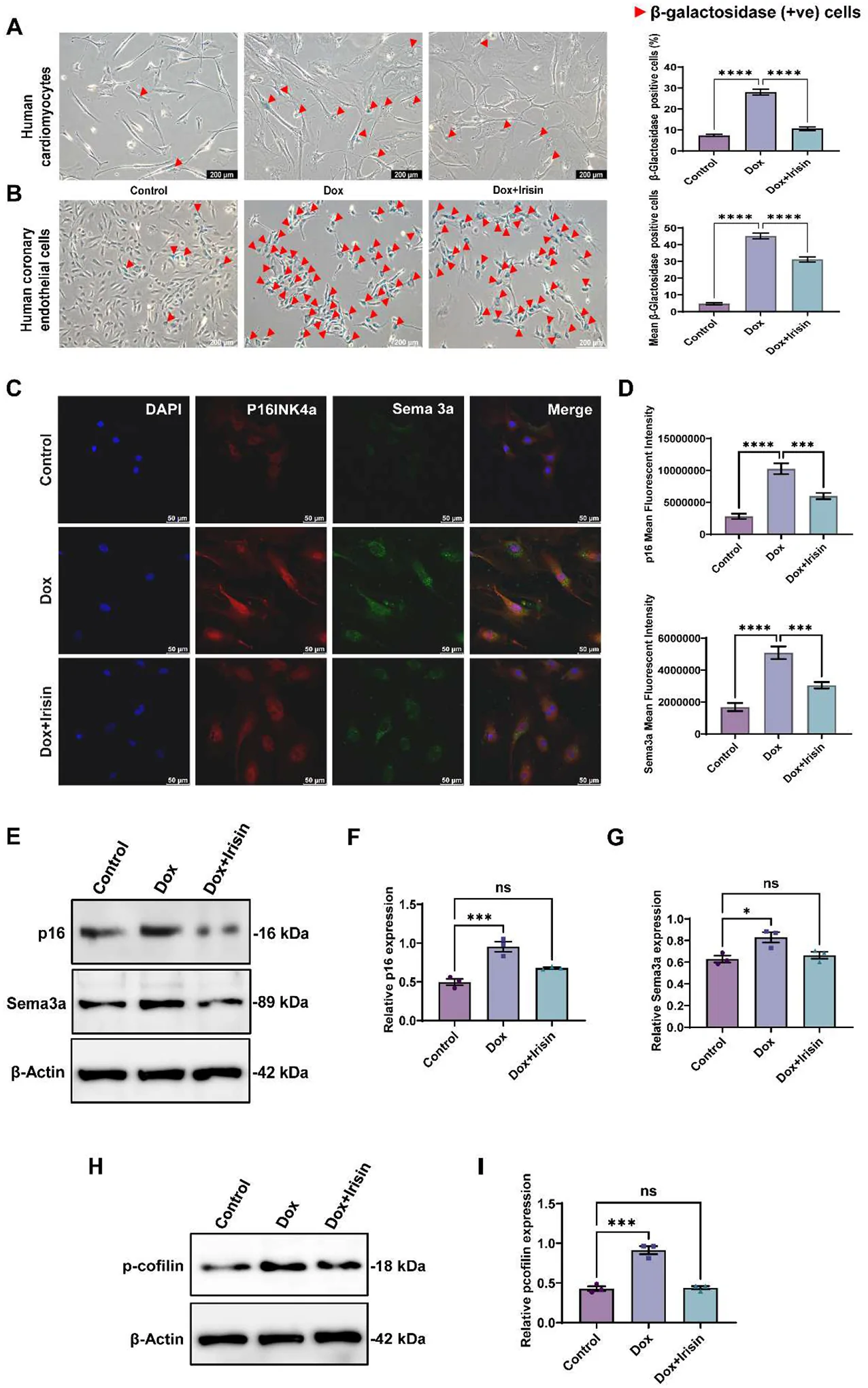
Irisin inhibits Dox-induced cardiac senescence (A, B) Representative β-Galactosidase activity in (A) human cardiomyocytes (HCMs) and (B) human coronary artery endothelial cells (HCECs) treated with Dox or Dox + irisin and their corresponding quantification. Scale bar = 200 μm. (C, D) Immunofluorescence analysis of p16 (red) and Sema3a (green) expression in control, Dox, and Dox+Irisin-treated HCEC with quantification of mean fluorescent intensity. Scale bar = 50 μm. (E-I) Western blot analysis of p16, Sema3a and p-cofilin expression in HCMs with densitometric analysis. Data are expressed as mean ± SEM; n=3. Statistical significance was determined using one-way ANOVA with multiple comparisons and significance is denoted by *p ≤ 0.05, ***p ≤ 0.001, and ****p ≤ 0.0001; ns, not significant.

**Fig. 6:**
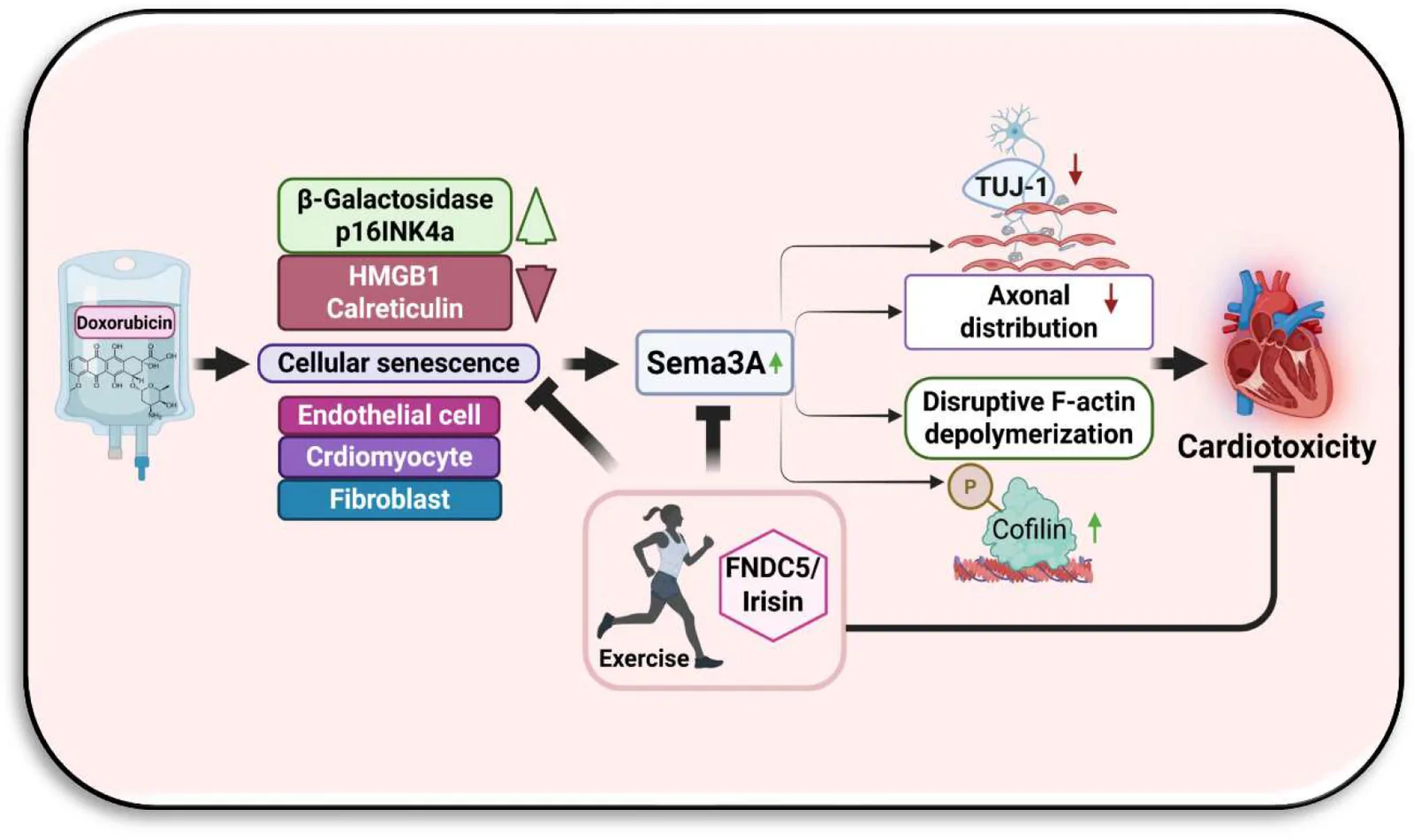
A schematic illustration depicts exercise-mediated protection against Dox-induced cardiac senescence and cardiotoxicity. Dox induces cellular senescence characterized by increased β-Galactosidase and p16^INK4a^ induction and the downregulation of HMGB1 and calreticulin with the subsequent upregulation of Sema3a and p-cofilin leading to F-actin disorganization, reduced axonal density (TUJ1) and progressive cardiac dysfunction. Exercise suppresses p16^INK4a^ and Sema3a expression, thereby attenuating cardiac senescence, and protecting against Dox-induced cardiotoxicity via FNDC5/irisin axis.
