# Supplementary material for: Exercise inhibits doxorubicin-induced cardiotoxicity by preventing cardiac and endothelial cell senescence by activating FNDC5/Irisin signaling
Source: Res Sq. 2026 Sep 29:rs.3.rs-10910107. Preprint. [Version 1] doi: 10.21203/rs.3.rs-10910107/v1 (PMC13648846; doi:10.21203/rs.3.rs-10910107/v1)

Supplementary figure legends:

Figure S1. Effect of Dox on cardiomyocyte senescence and cardiac function.

(A, B) Echocardiographic analysis of EF% (A) and FS% (B) in p16-3MR transgenic mice treated with Dox, n=4, *$p \leq 0.05$. (C) Immunofluorescence and quantitative analysis of p16-RFP in the hearts of control and Dox-treated p16-3MR transgenic mice. Data are mean ± SEM; n=5, Scale bar = 50 μm. Statistical significance was determined using a Student's t-test, *$p \leq 0.05$, **$p \leq 0.01$, ***$p \leq 0.001$. (D, E) β-Galactosidase staining and quantification of neonatal mouse (D) and human (E) cardiomyocytes treated with PBS or Dox. Arrows denote β-Galactosidase positive cells, a hallmark of cellular senescence. (F, G) Western blot analysis and relative protein expression showing decreased HMGB1 and calreticulin (senescence-associated proteins), in neonatal mouse cardiomyocytes (F) and human cardiomyocytes (HCMs) (G) following Dox treatment. Quantitative densitometry indicates fold changes relative to controls for each protein n=3, Scale bar = 200 μm. *$p \leq 0.05$, **$p \leq 0.01$, ***$p \leq 0.001$.

Figure S2. Exercise attenuates Dox-induced cardiac dysfunction.

(A) Representative M-mode echocardiography from parasternal long axis view in control, Dox, and Dox+Ex-treated mice, illustrating cardiac motion and wall dynamics. (B) Representative B-mode echocardiography from parasternal long axis view in control, Dox, and Dox+Ex mice. Videos

labelled with suprasternal view of the papillary muscle, right ventricle, left ventricle and aorta.

Supplementary S1

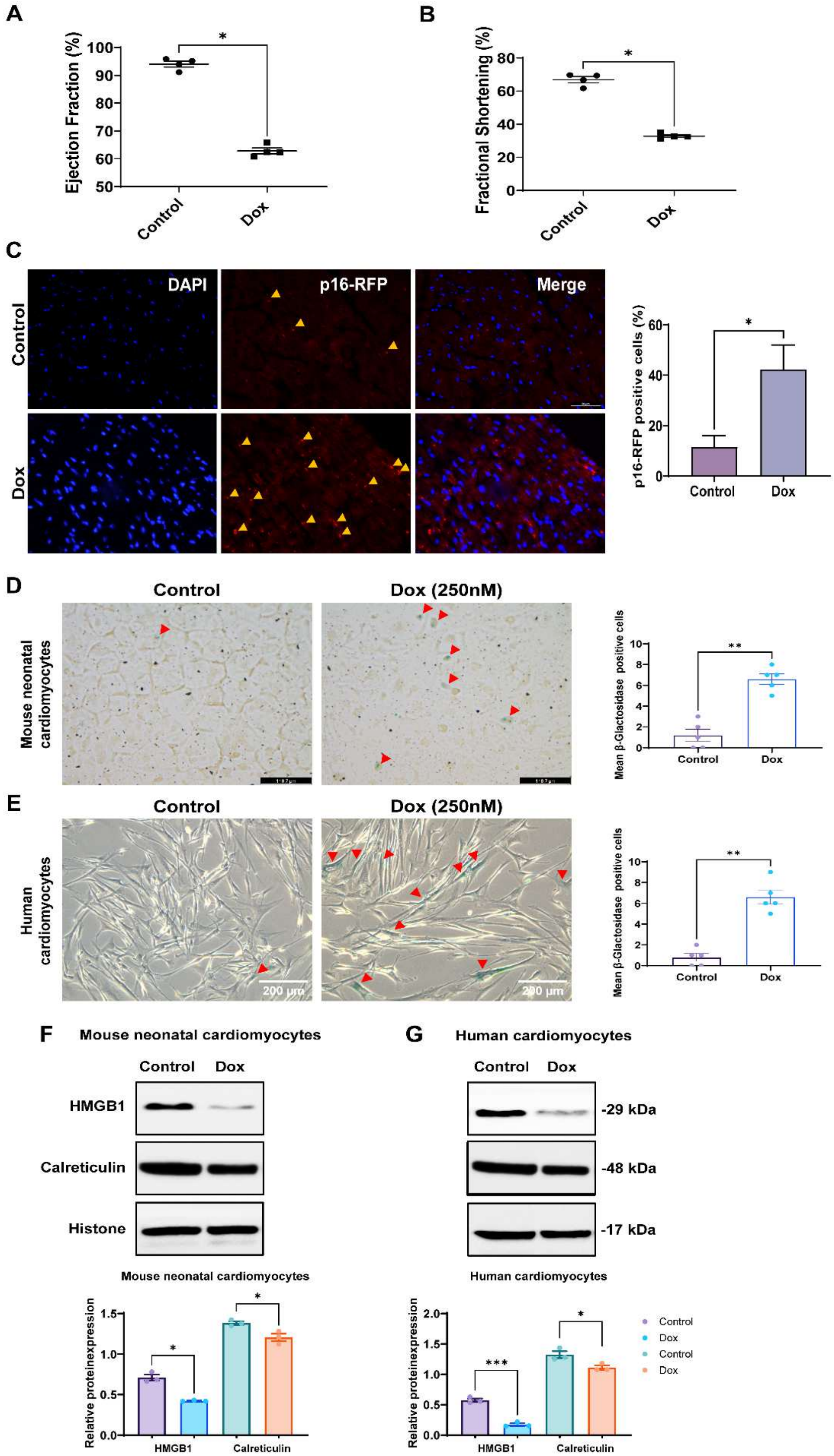

Supplementary S2

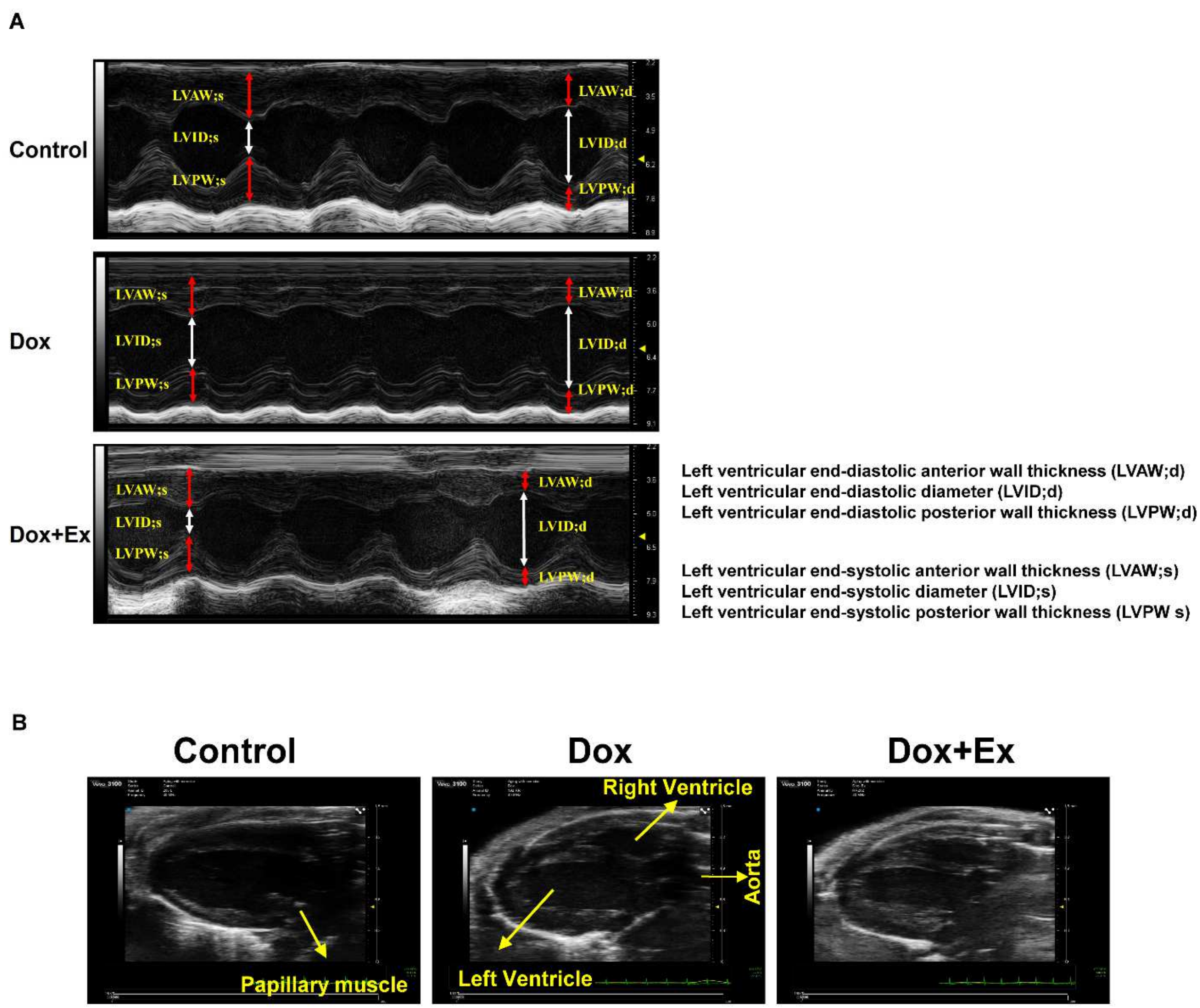

Supplement: Supplementary file 1 [file NIHPPRS10910107V1-supplement-1.pdf]
